# Intracellular Oxidative Stress Induced by Physical Exercise in Adults: Systematic Review and Meta-Analysis

**DOI:** 10.3390/antiox11091751

**Published:** 2022-09-04

**Authors:** Zhanyi Zhou, Chaoyi Chen, Ee-Chon Teo, Yan Zhang, Jialu Huang, Yining Xu, Yaodong Gu

**Affiliations:** 1 Faculty of Sports Science, Ningbo University, Ningbo 315211, China; 2 Savaria Institute of Technology, Faculty of Informatics, Eötvös Loránd University, H9700 Szombathely, Hungary

**Keywords:** exercise, oxidative stress, reactive oxygen species, redox, meta-analysis

## Abstract

A physical exercise program is one of the commonly used methods for improving an individual’s antioxidative capacity. However, an inappropriate physical exercise program would induce extra oxidative stress (OS), and the relationship between the details of a physical exercise protocol and the severity of intracellular OS is still unclear. A systematic review and meta-analysis of randomized controlled trials were conducted by searching PubMed, Medline, and Web of Science with the eligibility criteria: (1) participants over 18 years old; (2) physical exercise interventions; (3) 8-hydroxydeoxyguanosine, F2-isoprostanes, and protein carbonyls (PCs) as outcome measures; (4) published in English and peer-reviewed. 12 studies were included, and the data of 8 in them were pooled together. The agreement between authors reached a kappa value of 0.73. The results of the meta-analysis showed that: (1) the level of OS did not depend on the absolute intensity of physical exercise but on both the intensity and the volume of exercise; (2) high-intensity aerobic exercise (HIAE) and a combined protocol of HIAE and resistance training had the highest potential to induce large OS in unhealthy people; (3) the OS induced by moderate-to-high intensity aerobic exercise was significantly larger than that induced by ordinary life activities in healthy adults; (4) high-intensity interval training and moderate-intensity aerobic exercise had the lowest and sub-lowest probabilities to induce high intracellular OS for unhealthy adults. activities induce OS in various tissues in the human body, and the severity of OS depends on many factors of physical exercises as well as the health condition of an individual. A high-intensity and high-volume physical exercise program has the largest possibility of inducing severe OS, while a moderate-intensity aerobic exercise program and a high-intensity interval training program with a relatively low volume might be beneficial to the redox balance for unhealthy individuals. In conclusion, continuous aerobic exercise under moderate-intensity or high-intensity interval training could be recommended to enhance the body’s capacity for maintaining redox balance, especially for unhealthy individuals. The PROSPERO Registration Number is CRD42022349687.

## 1. Introduction

Oxidative stress is a part of the normal metabolic process, in which the cells continuously generate free radicals and nonradical derivatives of oxygen such as hydrogen peroxide (H_2_O_2_), superoxide, hydroxyl free radicals, and singlet oxygen. These chemical reactive molecules that contain oxygen are also called reactive oxygen species (ROS).

Biologically, ROS are highly reactive chemicals, the production mechanism of which is complicated [1]. Taking H_2_O_2_, for example, the reduction of molecular oxygen (O_2_) produces superoxide (O_2_^●−^), which is the precursor to most other ROS. Then, the O_2_^●−^ dismutates and produces hydrogen peroxide (H_2_O_2_). Finally, H_2_O_2_ will in turn be partially reduced by forming hydroxide ions and hydroxyl radicals (^•^OH) or fully reduced to water [2]. This pathway is the main source of H_2_O_2_ in cells. At the same time, O_2_^●−^ and nitric oxide (also belongs to ROS) can trigger chemical reactions to form other kinds of ROS.

Although ROS are by-products of the normal oxygen-related metabolism and have roles in cell signaling and homeostasis [3,4,5], they could induce tissue oxidative damage in the absence of antioxidants [6]. For human beings, ROS accumulate with the energy metabolism related to physical activities and induce oxidative stress when their production exceeds the neutralization capacity of human tissues [7,8]. The mechanism of the oxidative stress induced by ROS is that ROS trigger strong oxidation reactions in human cells and destroy cells by their strong oxidation. Generally, the harmful effects of oxidative stress on the cells of the human body are the damage of DNA or RNA, the oxidations of polyunsaturated fatty acids in lipids, which is also called lipid peroxidation, the oxidations of amino acids in proteins, and the oxidative deactivation of specific enzymes by the oxidation of co-factors [9].

For now, physical exercise has been one of the major methods to improve the body’s health [10,11]. However, the evidence shows a contradictory trend in the discussion about the effect of physical exercise on the dynamic redox balance. Although various physical exercise programs seem to have a positive effect on antioxidation [12], some studies have also found that physical exercise will cause greater metabolic and oxidative stress in the human body, which could be detrimental to health. For example, it has been verified that physical activities would induce the production of ROS and then induce oxidative stress, which has a negative effect on the function of skeletal muscles [13]. It also has been demonstrated that ROS will cause premature fatigue during exercise [14].

The contradiction in the evidence might come from the heterogeneity of the study designs and protocols such as the different intensities of the physical exercise interventions and the population of the participants in the trials [15,16]. For example, in 2006, a study by Dékány’s team found that the redox balance of athletes had a relationship with their physical status [17]. Another study published in 1999 demonstrated that there was no difference between the effect of physical activity under moderate intensity on oxidative stress for pre-menopausal and post-menopausal women [18]. In 2017, Jemili’s team identified that a 3-month specific training program could improve the redox balance of elite karate athletes and should be recommended for athletes with similar physical fitness levels [19].

Meanwhile, the measurement of oxidative stress in human tissues is very difficult and complex since, in the process of exercise, the intensity of oxidation reaction in different human tissues is different, and the ROS are so chemically active that they could only be detected when all the antioxidants have been depleted in tissues [20]. Some systematic reviews have identified the oxidative stress induced by physical exercise by using blood parameters as outcome measures. For example, a systematic review and network meta-analysis published in 2022 identified the effect of physical exercise under different intensities and antioxidative supplementation for plasma superoxide dismutase (SOD) in healthy adults, claiming that an exercise program under a specific intensity will induce different severities of oxidative stress for healthy individuals with different daily activities. Moreover, a systematic review published in 2021 by Ye’s team demonstrated that regular aerobic exercise could significantly reduce blood oxidant markers including malondialdehyde (MDA) and lipid peroxide (LPO) and increase the levels of antioxidant factors such as nitric oxide (NO), superoxide dismutase (SOD), and total antioxidant capacity (TAC) in elder adults [21].

The balance between oxidation and antioxidation is key to maintaining muscle function and reducing muscle soreness. However, analyzing oxidative stress only through indicators of blood is not enough for understanding the overall oxidative stress condition of an individual. To understand the overall oxidative stress condition of an individual, it is also necessary to analyze the intracellular oxidative stress. At present, there is a lack of sufficient high-quality evidence for exercise-induced oxidative stress in human cells. Therefore, it is necessary to identify the intracellular oxidative stress induced by different physical exercise programs without the interference of antioxidative supplementation.

The intracellular oxidative stress related to exercise is usually analyzed by measuring the reactive metabolites of the ROS of different parts of human cells, since the reactive metabolites of ROS are chemically more stable than ROS. The oxidative product of DNA is usually measured by the radical modification of guanine in tissue, plasma, and urine, a common indicator of which is 8-hydroxydeoxyguanosine (8-OHDG) [22]. MDA, lipid hydroperoxides (LOOH), and F2-isoprostanes are commonly applied in the quantification of lipid oxidation, among which the LOOH and F2-isoprostanes are both derived from lipids in cytomembranes, and F2-isoprostanes is considered to be a superior indicator of lipid peroxidation [23,24]. The protein oxidation is quantified by measuring protein carbonyls (PCs) formation and could be sampled from skeletal muscles or plasma [25].

The objective of this systematic review is to identify the oxidative stress induced by physical exercise in human cells by comparing the oxidative indicators of membrane, DNA, and protein.

## 2. Methods

### 2.1. Eligibility Criteria

#### 2.1.1. Participants

Trials with participants over 18 years old and without any musculoskeletal disease or clinical diagnosis of exercise contraindication were eligible for this systematic review. The studies were reclassified into two subgroups according to the health conditions of their participants: (1) the unhealthy subgroup referred to studies whose participants were individuals with clinically diagnosed diseases; (2) the healthy subgroup referred to studies whose participants were individuals without clinically diagnosed diseases. It was necessary to emphasize that studies whose participants were obese, overweight, and sedentary individuals were reclassified into the healthy subgroup.

#### 2.1.2. Interventions

This systematic review included trials that asked their participants to conduct physical exercise programs as interventions. All interventions were classified into the following categories according to their intensities and types of movements: (1) moderate-to-high-intensity (or vigorous) aerobic exercise, with the abbreviation being “MHAE”; (2) moderate-intensity aerobic exercise, with the abbreviation being “MIAE”; (3) high-intensity aerobic exercise, with the abbreviation being “HIAE”; (4) high-intensity interval training, with the abbreviation being “HIIT”; (5) resistance training, with the abbreviation being “RT”; (6) strength training, with the abbreviation being “ST”. If an intervention combined more than one of the categories above, a plus sign was used to connect the abbreviations. For example, the abbreviation “MIAE + RT” meant a combination of moderate-intensity aerobic exercise and resistance training.

#### 2.1.3. Comparators (C)

Trials whose participants in control groups were asked to maintain their current lifestyle or maintain current physical activity and were allowed to add some gentle activities such as stretching or relaxation were included in this systematic review, with the abbreviation being “MCA”. Moreover, all the interventions in the experimental groups were also regarded as comparators.

#### 2.1.4. Outcomes (O)

This systematic review included trials whose outcome measures were the oxidative stress indicators in the membrane, protein, and DNA of human cells. The laboratory samples of the outcome measures could be urine, blood, or cell biopsy samples. The oxidative stress indicators of the membrane, DNA, and protein were F2-isoprostanes, 8-hydroxydeoxyguanosine (8-OHDG), and protein carbonyls (PCs).

#### 2.1.5. Study Design (S)

Only randomized controlled trials were included in this systematic review.

#### 2.1.6. Exclusion Criteria

Trials were excluded if: (1) participants had musculoskeletal diseases or were clinically diagnosed with exercise contraindication; (2) there were participants below the age of 18; (3) participants were asked to have antioxidative supplementations, drugs, or injections during the process of intervention; (4) the trial was a published abstract without full text or there was a lack of data; (5) outcome measures did not correspond with those in the eligibility criteria.

### 2.2. Information Sources

A comprehensive and reproducible search was performed on the PubMed, Medline, and Web of Science databases from January 2000 to May 2022. The studies must have been peer-reviewed and published in English. Reference lists of included studies were also searched. Grey literature was searched to identify potential studies. If the data were insufficient, the authors would be contacted and asked for the missing data.

### 2.3. Search Strategy

The Boolean logic searching was conducted according to the following principles: (1) have “exercise” or “training” in the title; (2) have “randomized” or “randomised” in the abstract; (3) have “8-OHDG” or “8-hydroxydeoxyguanosine” or “PCs” or “protein carbonyls” or “F2-isoprostanes” in the abstract; (4) without “review” or “design” or “protocol” in the title.

Two independent authors (Zhanyi Zhou and Chaoyi Chen) screened all the titles of the searched trials to identify all the potential trials before the abstract screening. A third independent librarian (Gusztáv Fekete) was invited to support the search strategies, checking other synonyms and entry terms to increase their sensitivity and specificity.

### 2.4. Selection Process

Trials that were searched from the databases were imported into EndNote 20 (Thomson Reuters, Carlsbad, CA, USA) to further screen and remove duplicates. Further screening was performed by two independent authors (Zhanyi Zhou and Yan Zhang). Any disagreement was resolved by a third independent author (Jialu Huang).

### 2.5. Data Collection Process

The data were collected by two independent authors (Zhanyi Zhou and Ee-Chon Teo). An independent reviewer was invited to check all the collected data (Yining Xu).

### 2.6. Data Items

The following information was collected and recorded: (1) The characteristics of participants, such as the health condition, average age, and gender ratio; (2) Information about the intervention programs, such as the names of the interventions, details of the programs, and their categories; (3) Outcome measure results, such as the sample size of each group, the recording time, the units, and the mean value with its standard deviation in each record time.

### 2.7. Study Risk of Bias Assessment

The Cochrane Collaboration Risk of Bias Assessment Tool was used to evaluate the risk of bias in individual studies [26]. According to the Cochrane Collaboration Risk of Bias Assessment Tool, a study with no item with high risk and fewer than three (contain) items with unclear risk was regarded as having an overall low risk; a study with no item with high risk and more than three items with unclear risk was regarded as having an overall moderate risk, a study had one item with high risk was also regarded as having an overall moderate risk; and a study with more than one item with high risk was regarded as having an overall high risk. Agreement between authors was determined by Cohen’s kappa value. Two independent authors (Zhanyi Zhou and Chaoyi Chen) assessed all of the included studies. An independent arbitrator (Ee-Chon Teo) was invited when an agreement could not be met.

### 2.8. Effect Measures

Since the units of outcome measures in different trials might be various, in the systematic review, the effect was presented in the form of standardized mean differences and their standard error (SMD ± SE).

The effect size of the pair-wise meta-analysis was presented in the form of standard mean difference (SMD). According to the criteria suggested by Cohen, the effect size was regarded as large when the SMD was larger than 0.8, moderate when the SMD was from 0.5 to 0.8, small when the SMD was from 0.2 to 0.5, and very small when the SMD was less than 0.2 [27].

### 2.9. Synthesis Methods

#### 2.9.1. Study Information Synthesis

All the data items collected were input in a table, as well as the main conclusion about the oxidative stress indicators provided by each included study. The original data of each outcome measure are provided in the Supplementary File Table S1.

#### 2.9.2. Data Pre-Processing

Data pre-processing was performed by two independent investigators (Ee-Chon Teo and Chaoyi Chen). Microsoft Office Excel (Version 16.0, Microsoft Corporation, Redmond, WA, USA) was used to pre-process the original data. All the outcome measures were converted into standardized mean differences in each recording time. Moreover, the effect sizes of changes in overall oxidative stress and changes in individual indicators were calculated separately. If a trial reported more than one indicator of oxidative stress, the average standard mean difference and its standard error were calculated by the following formula:

(1) $$SMD_{ave}=\frac{1}{2}\left(SMD_{1}+SMD_{2}\right)$$

(2) $$SE_{SMD}=\sqrt{SMD_{1}^{2}+SMD_{2}^{2}}$$

#### 2.9.3. Data Synthesis

A geometry of intervention comparisons, which was made by the Confidence in Network Meta-Analysis (CINeMA https://cinema.ispm.unibe.ch, accessed on 26 July 2022), was applied to show the evidence structure. The geometry provided key information about evidence structure: (1) the risk of bias distribution of each intervention was represented by the color in every node (Red = high risk of bias, Yellow = unclear risk of bias, Green = low risk of bias); (2) the sample size of each intervention was represented by the size of the node; (3) the arms of each direct comparison was represented by the width of lines that connected two interventions.

If there were only two interventions in the evidence structure of comparison, a pair-wise meta-analysis was applied to synthesize the data, while, if there were more than two interventions in the evidence structure of comparison, a network meta-analysis was applied. The Review Manager (Version 5.3, Cochrane Collaborate, John Wiley & Sons, Inc., Hoboken, NJ, USA) was used to make the pair-wise meta-analysis. The trials with different follow-up durations in the included trials were separated into independent trials according to their endpoints. The pooled effect was presented in a total form by a forest plot with the effect size and its *p*-value, the heterogeneities within studies were assessed by the I-square, and the existence of publication bias was descriptively presented by a funnel plot [27]. Moreover, to identify the source of heterogeneities within studies, subgroup analyses were conducted according to the age of the participants and the different oxidative stress indicators of the membrane, protein, and DNA. The results of the subgroup analyses were presented in a subtotal form by a forest plot with the effect size and its *p*-value, and the heterogeneities within studies were assessed by the I-square.

The network meta-analysis was conducted by the Aggregate Data Drug Information System (ADDIS V1.16.8, http://drugis.org/software/addis/index, accessed on 30 July 2022).

### 2.10. Reporting Bias Assessment

According to The Cochrane Collaboration Risk of Bias Assessment Tool, if the included study had a pre-registered protocol number and all the outcomes in the protocol were fully matched with those reported in the article, this study was regarded to have a low risk of selective reporting. Meanwhile, if the included study had a pre-registered protocol number but the outcomes reported in the article were not fully matched with those registered in the protocol, this study was regarded as having a high risk of selective reporting. At last, if the included study did not have a pre-registered protocol number, this study was regarded as having an unclear risk of selective reporting [26]. The results of the bias assessment were provided in the risk of bias assessment results.

### 2.11. Certainty Assessment

The Confidence in Network Meta-Analysis (CINeMA https://cinema.ispm.unibe.ch, accessed on 30 July 2022) was used to evaluate the confidence and assess the reporting bias in the findings from the network meta-analysis [28,29]. According to the method research of CINeMA [29], if the item “within-study bias” was of “Major concern”, the confidence should be downgraded by one level, whereas if other items were of “Some concern”, the confidence would be downgraded by one level, and if they were of “Major concern”, the confidence would be downgraded by two levels.

## 3. Results

### 3.1. Study Selection

**Figure 1:**
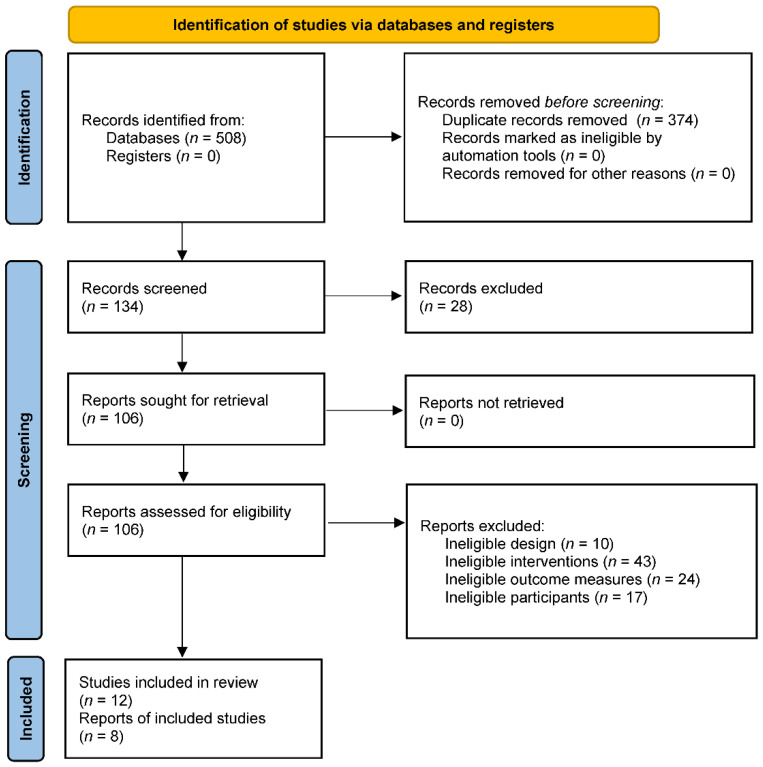
The flow diagram of study selection.

The search yielded 508 titles and abstracts for screening. A total of 374 duplicated studies were removed, and 134 studies were included in the records screening. Then, 28 studies that were not RCTs were excluded, and 106 studies were included for full-text screening. Among the 106 studies, 10 studies were excluded due to their ineligible design, 43 studies were excluded because of their ineligible interventions, 17 studies were excluded because of their ineligible participants, and 24 studies were excluded because of their wrong outcome measures. Eventually, 12 studies were included in the systematic review [30,31,32,33,34,35,36,37,38,39,40,41], and, except for 6 studies that lacked original data, 8 studies were included in the final analysis [30,31,32,34,35,38,39,41]. The flow diagram is presented in Figure 1.

### 3.2. Study Characteristics

According to the results of the study selection, six studies reported oxidative stress induced by physical exercise in healthy adults, while six studies reported the same thing in unhealthy adults. Four studies included participants over 60 years old, eight studies included participants younger than 60 years old, and three studies included young participants. Detailed information about all the included studies is provided in Table 1.

### 3.3. Risk of Bias in Studies

**Figure 2:**
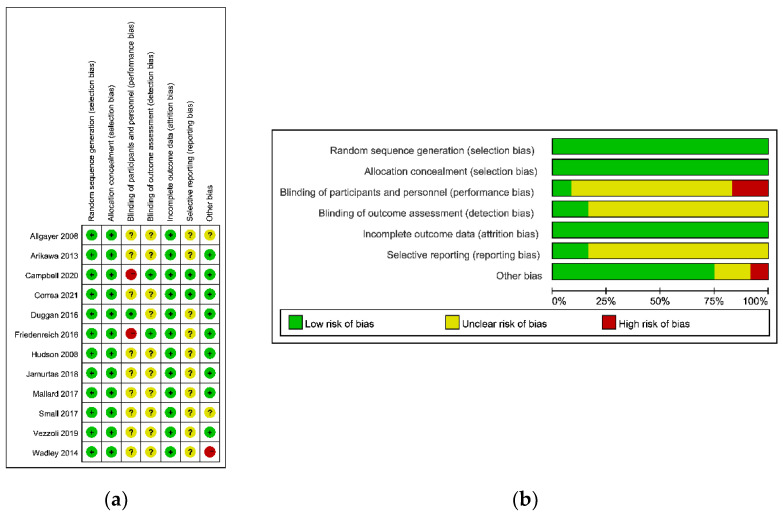
The risk of bias assessment. (**a**) Risk of bias summary; (**b**) risk of bias graph.

A consensus was reached for all items, and the agreement between authors reached a kappa value of 0.73. The result of the risk of bias assessment is shown in Figure 2. According to the results, no study had a high risk of bias, five studies had a moderate risk of bias, and seven trials had a low risk of bias.

### 3.4. Results of Individual Studies

The results of individual studies are presented in Table 2.

### 3.5. Results of Syntheses

#### 3.5.1. Evidence Structure

**Figure 3:**
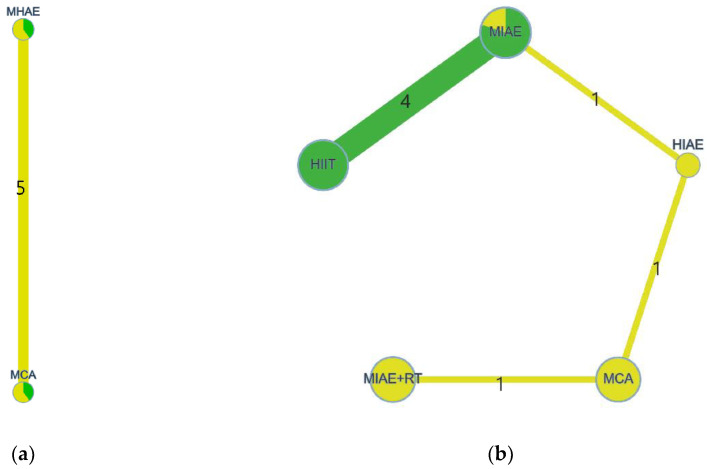
The evidence structure of interventions. (**a**) Healthy participants; (**b**) unhealthy participants.

Eight included studies provided the original data of their outcome measures and were input into the data synthesis. The evidence structure was presented in Figure 3. In Figure 3, the risk of bias distribution of each intervention was represented by the color in every node (Red = high risk of bias, Yellow = unclear risk of bias, Green = low risk of bias), the sample size of each intervention was represented by the size of the node, and the arms of each direct comparison were represented by the width of the lines that connected two interventions and the numbers on them.

According to Figure 3, the evidence structure of intracellular oxidative stress induced by physical exercise in healthy adults had only two interventions, which were MHAE and MCA. Therefore, a pair-wise meta-analysis was applied to calculate the pooled effect. At the same time, the evidence structure of intracellular oxidative stress induced by physical exercise in unhealthy adults had more than two interventions. Therefore, a network meta-analysis was applied.

#### 3.5.2. Pair-Wise Meta-Analysis

**Figure 4:**
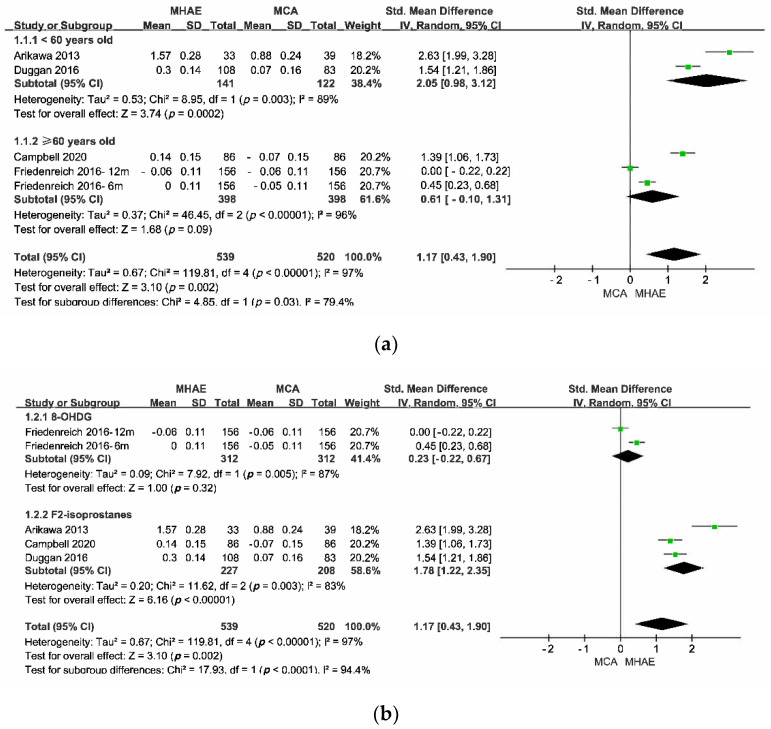
The results of the pair-wise meta-analysis. (**a**) Divided by the age of participants; (**b**) divided by outcome measures.

To identify the source of heterogeneities within studies in the pair-wise meta-analysis, subgroup analyses were conducted according to the age of the participants and the different oxidative stress indicators of the membrane, protein, and DNA. The results of the total and subtotal are presented in Figure 4. According to Figure 4, the pooled effects showed that MHAE could induce more oxidative stress when compared with MCA, and the difference reached a large effect size (Z = 3.10) with statistical significance (*p* < 0.00001). According to the results of the subgroup analyses, the pooled effect of the oxidative stress induced by MHAE in healthy adults younger than 60 years old was significantly larger than that induced by MCA (Z = 3.74, *p* = 0.0002), while the pooled effect in the subgroup of elders (>60 years old) favored MHAE but did not reach a significant difference (Z = 1.68, *p* = 0.09). When it came to the subgroup analysis, which was divided by outcome measures, the oxidative stress induced by MHAE in DNA, whose outcome measure was 8-OHDG, was larger than that induced by MCA (Z = 1.00) but did not reach a statistical significance (*p* = 032), while the intracellular oxidative stress induced by MHAE in the membrane, whose outcome measure was F2-isoprostanes, was significantly larger than that induced by MCA (Z = 6.16, *p* < 0.00001).

**Figure 5:**
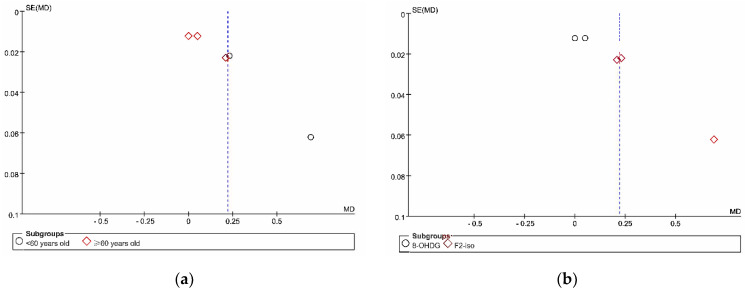
The funnel plots of the pair-wise meta-analysis. (**a**) Divided by the age of participants; (**b**) divided by outcome measures.

The total heterogeneity within studies was large (I^2^ = 0.97), while the heterogeneities in a subgroup or between subgroups were large (I^2^ > 75%). The description of publication bias was represented by funnel plots, as in Figure 5. According to Figure 5, the pair-wise meta-analysis might have an obvious publication bias since the funnel plots of the subgroup analyses were asymmetric.

The publication bias within each subgroup analysis is descriptively shown by the funnel plots in Figure 5. According to the funnel plots, the points were not symmetrically distributed on either side of the dotted lines, indicating that there might be publication bias in both subgroup analyses.

#### 3.5.3. Network Meta-Analysis

**Figure 6:**
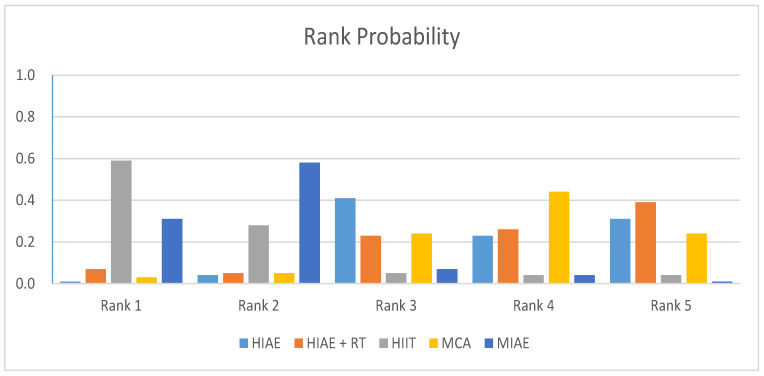
The probability rank of each intervention for unhealthy participants.

The random-effects standard deviation of consistency was 1.40 (95%CI: 0.07 to 2.83), while that of the inconsistency model was 1.50 (95%CI: 0.04 to 2.83). Since the random-effects standard deviations calculated under consistency and inconsistency models were fully identified and the evidence structure had no closed loop, the consistency model was appropriate to conduct the network meta-analysis. Figure 6 and Table 3 provided the probability rank of every intervention, in which a lower rank number meant the induction of lower oxidative stress. Therefore, in the probability rank of interventions, the intervention in Rank N would induce higher oxidative stress.

According to the rank of probability presented in Table 3, a combination program of high-intensity aerobic exercise and resistance training had the most potential to induce high intracellular oxidative stress in unhealthy adults (0.39 in Rank 5), a high-intensity aerobic exercise program had the sub-most potential to induce high intracellular oxidative stress in unhealthy adults (0.31 in Rank 5), and a high-intensity interval training program and moderate-intensity aerobic exercise had the lowest and sub-lowest probabilities to induce high intracellular oxidative stress in unhealthy adults (0.59 in Rank 1 and 0.58 in Rank 2).

### 3.6. Reporting Bias

The reporting bias is reported in Figure 2. According to Figure 2, only 2 included studies had a low risk of selecting reporting, while the other 10 included studies did not pre-register their trials. Therefore, although there was no included study with a high risk of reporting bias, the overall reporting bias of the systematic review was moderate, since 83.3% of the included studies reported an unclear risk of selective reporting.

### 3.7. Certainty of Evidence

**Figure 7:**
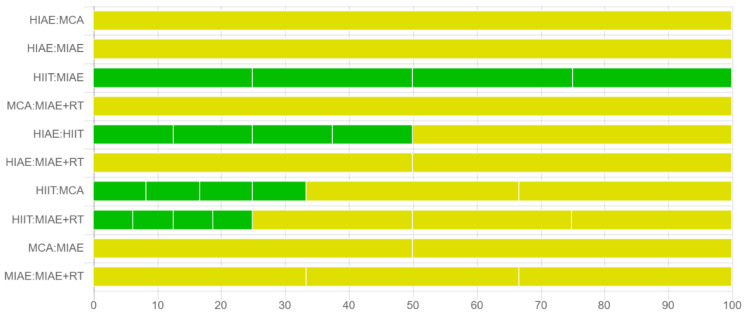
Contribution to the average risk of bias of each pair intervention comparison.

Figure 7 shows the contribution to the average risk of bias of each pair intervention comparison, while Table 4 provided the results of the confidence assessment made by CINeMA.

In Table 4, the “MC” with red color means the issue needs major concern, the “SC” with yellow color means the issue needs some concern, while the “NC” with green color means the issue needs no concern. According to Table 4, the confidence ratings of all the direct and indirect comparisons were very low, and the most common reasons for the downgrade were incoherence, reporting bias, and heterogeneity.

## 4. Discussion

The objective of this systematic review is to compare the oxidative stress induced by physical exercise in human cells. Through comparing the oxidative indicators of the membrane, DNA, and protein, the main findings are as follows. First, according to the results of individual studies, different physical exercise protocols induce different levels of oxidative stress, and the oxidation seems to occur in all parts of human cells, regardless of whether the individual is healthy or not. Second, the level of oxidative stress seems not to depend on the absolute intensity of physical exercise but depends on both the intensity and the volume of physical exercise, since HIAE and HIAE + RT have the highest potential to induce large oxidative stress in unhealthy people, and the oxidative stress induced by MHAE is significantly larger than that induced by MCA in healthy adults. Last, HIIT and MIAE might improve the antioxidation ability of the human body since they have the lowest and sub-lowest probabilities to induce high intracellular oxidative stress in unhealthy adults (0.59 in Rank 1 and 0.58 in Rank 2).

Corresponding to the results of previous studies, physical activities induce the generation of ROS, which will cause oxidative stress in various tissues in the human body, and the severity of oxidative stress depends on the protocol of physical exercises and the healthy condition of individuals. However, although this systematic review excluded interventions that contain antioxidant supplementation, making it possible to explore the independent effect of physical exercise on oxidative stress in human cells, what should not be ignored is the different abilities of individuals in adjusting the balance between oxidation and antioxidation. For example, a systematic review and meta-analysis published in 2020 identified that, in young adults, there would be significant alterations in oxidative stress and cytokine levels after acute exercise, ranging from moderate to extremely large, while the variations after chronic exercise ranged from trivial to moderate. However, this systematic review had an obvious publication bias and high heterogeneity, which might downgrade the confidence of the pooled evidence [42]. At the same time, in another systematic review and meta-analysis published in 2020, the participants, including trained and untrained individuals, claimed that there would be a substantial increase in DNA oxidative damage occurring immediately following acute aerobic exercise [43]. Further studies should explore the oxidative stress induced by a specific exercise program in populations with different abilities of antioxidation and nutrition statuses.

The results of the pair-wise meta-analysis and network meta-analysis indicate that the severity of oxidative stress induced by physical exercise not only depends on the intensity but also the total volume of the exercise program. The pooled effect of the pair-wise meta-analysis shows that moderate-to-high-intensity aerobic exercise would induce larger intracellular oxidative stress in healthy adults, while the rank probabilities of interventions in the result of the network meta-analysis show that high-intensity aerobic exercise or a combination of high-intensity aerobic exercise and resistance training might induce more oxidative stress than other physical exercise protocols for unhealthy adults. This finding is different from the results of some previous studies, which claim that a higher severity of oxidative stress is correspondent with a higher intensity of physical activity [14,44]. The biomarkers of oxidative stress only appear after the antioxidant is exhausted in cells and must be measured at the right time and in the right position of the body [20]; therefore, the potential mechanism is possibly that, although high-intensity physical activity can generate larger oxidative stress, the antioxidants in the cells cannot be exhausted when the total volume of activity is relatively small. Therefore, the biomarkers for oxidative stress would not be generated, and the reactive metabolites of ROS would not be detected from relevant tissue samples in the laboratory. This hypothesis has been identified by some previous studies. For example, in 2010, a study conducted by Bloomer’s team found that a long-term and high-volume aerobic exercise program induced the balance of redox balance towards the antioxidative direction [45], and a comparative study published in 2018 demonstrated that a long-term and high-intensity program induced significant oxidative stress and increased the indices of skeletal muscle damage in both post-pubertal girls and boys [46]. Another study published in 2004 identified that higher-intensity (load) resistance training would induce significantly less oxidative stress and muscle disruption because, within a specific duration, a resistance training plan with a larger load means fewer repetitions, a longer interval rest, and relatively less volume [47].

Another primary finding from the results of the network meta-analysis is that a moderate-intensity aerobic exercise program and high-intensity interval training might be beneficial to the redox balance for unhealthy individuals because of their lower possibilities of inducing severe oxidative stress. This finding is also correspondent with the conclusions of some previous studies. For instance, a study published in 2021 by Lu’s team identified that a high-intensity interval training program could enhance the body’s antioxidant system, letting individuals suffer less oxidative stress after physical activities, and suggested that, for untrained humans, having daily exercise under intensities above 70% VO_2max_ would be proposed for initial exercise levels [48]. A study conducted by Lope’s team in 2016 demonstrated that a moderate-intensity exercise program could be beneficial to attenuating the susceptibility of oxidative damage and to increasing endothelial function [49]. As has been illustrated above, the rate of generation of oxidative stress induced by moderate-intensity exercise might not be high enough to exceed the capacity of antioxidation in cells, while the absolute volume of a high-intensity interval training program might be relatively small to create enough ROS to deplete the antioxidants in body cells. This hypothesized mechanism has also been partially verified by previous studies. In 2013, a study investigated the changes in oxidative stress biomarkers and antioxidant status indices caused by a short-term HIIT program and found that a short-term HIIT program could attenuate oxidative stress responses and improve antioxidant status in healthy adults [50]. Moreover, a study by Tucker’s team concluded that a HIIT program was superior to sedentary behavior and low-intensity exercise since it could beneficially influence the expression of genes related to endogenous antioxidant enzyme activity and then reduce the severity of oxidative stress [51]. Nevertheless, the heterogeneity in the protocol of HIIT, such as the intensity, interval resistance, and total volume, means this hypothesis must be confirmed in further studies.

However, what should not be ignored is the heterogeneity induced by the different clinical populations of the participants in studies that included the unhealthy subgroup. The studies in the unhealthy subgroup have participants with rheumatoid arthritis, kidney disease, type 2 diabetes, colorectal cancer, and sarcopenia; therefore, the redox balance conditions before the intervention of these participants with different diseases were heterogeneous. This is why there are still contradictions between the pooled effect of this review and the results of other related clinical studies. Taking type 2 diabetes, for example, only one included study reported exercise-induced oxidative stress for type 2 diabetes patients, claiming that no significant difference was found within the oxidation of lipids and proteins between HIIT and MIAE, which was correspondent with the pooled results of the network meta-analysis [38]. However, some previous studies have presented different views on the role of exercise in oxidative stress in patients with type 2 diabetes. A study published in 2008 investigated whether low-intensity exercise can modulate the transcription factor peroxisome proliferator-activated receptor gamma (PPARgamma), finding that low-intensity exercise could increase PPARgamma DNA-binding activity [52]. Moreover, studies published in 2012 and 2015 both demonstrated that exercise-triggered monocyte PPARgamma activation might be the mechanism of the benefit created by exercise for patients with type 2 diabetes or related cardiovascular complications [53,54]. This might be the most important limitation of this systematic review, indicating that, on one hand, for clinical populations with different underlying diseases, the indicators to identify their intracellular oxidative stress may be different from those of healthy people and should be selected according to the special effects of their diseases on oxidative metabolism. On the other hand, more high-quality studies are also needed so that future reviews can perform higher-quality subgroup analyses based on clinical populations with different underlying diseases.

This systematic review has some other limitations that must be noted. First, the heterogeneities within studies and subgroups are large, and the source of heterogeneities is unclear, making the strength of the pooled evidence of this systematic review lower than expected. The differences in intervention protocols, participants, and study designs can all be the source of heterogeneities. However, since there are not enough studies included, it is infeasible to conduct a meta-regression to identify the specific cause of heterogeneities. Second, there is no official standard for measuring oxidative stress indicators. This systematic review included 8-OHDG, F2-isoprostanes, and PCs as outcome measures. However, these biomarkers are only metabolites of ROS reactions and could not represent the overall oxidative stress condition in the body, since other biomarkers can be used in measuring oxidative stress. For example, lipid hydroperoxides (LOOH) are other biomarkers that represent the oxidation of the membrane, and malondialdehyde (MDA) is also one of the biomarkers of cell lipid oxidation. Last but not the least, the nutritional condition of the body will affect the antioxidative mechanism in cells, especially the antioxidant. Therefore, the clinical value of this systematic review only considered the overall clinical health status of the participants and did not consider the underlying differences in the nutritional status of the participants.

## 5. Conclusions

In conclusion, this systematic review holds that, on the one hand, if an individual does not take any antioxidant supplements, he or she should pay attention not only to the intensity of physical exercise but also to the overall volume of exercise to avoid the damage of body tissue caused by oxidative stress. On the other hand, continuous aerobic exercise under moderate-intensity or high-intensity interval training could be recommended to enhance the body’s ability to maintain oxidative balance, especially in unhealthy individuals.

## 6. Other Information

This review was conducted according to the Preferred Reporting Items for Systematic Reviews and Meta-Analysis guidelines (PRISMA 2020) [55]. Literature screening criteria and study search strategies were proposed and agreed upon by two independent authors (Zhanyi Zhou and Chaoyi Chen). The PROSPERO Registration Number of this systematic review and meta-analysis is CRD42022349687.

## Figures and Tables

**Table 1:** Study Characteristics.

Study	Participants			Interventions				
	Population	Average Age	Gender (F/M)	Duration	Group	Description	Protocol	Category
Friedenreich 2016 [35]	Inactive, healthy, postmenopausal women	60.9	320/0	12 months	Experimental Group	Moderate-to-vigorous aerobic exercise	45 min/per time, from 50–60% HRmax to 70–80% HRmax, 225 min/week	MHAE
					Control Group	Maintaining current active levels	Maintaining current active levels	MCA
Vezzoli 2019 [40]	Elders with sarcopenia	72.4	19/16	12 weeks	Experimental Group	Moderate-intensity resistive exercise training	3/week, 6–8 min aerobic warm-up, 3 sets of 14–16 reps of chest presses, horizontal leg-presses, vertical rows, and shoulder exercises with free weights, 60% 1RM.	RT
					Control Group	Maintaining current active levels	Maintaining regular activities of daily living	MCA
Correa 2021 [33]	Hypertensive patients with stage II chronic kidney disease	58.0	23/37	6 months	Experimental Group	Resistance training	3/week, 3 sets of 12 reps at 50% 1RM, 3 sets of 10 reps at 60% 1RM, 3 sets of 8 reps at 70% 1RM.	RT
					Control Group	Maintaining current active levels	Maintaining current active levels	MCA
Hudson 2008 [36]	Adults with resistance training experience	21.8	Unclear	1 time	Experimental Group	Hypertrophy resistance training	4 sets, 10 reps with 90 s of rest at 75% 1RM	RT
					Control Group	Strength training	11 sets, 3 reps with 5 min of rest at 90% 1RM	ST
Jamurtas 2018 [37]	Healthy young men	22.4	0/12	1 time	Experimental Group	High-intensity interval training	4 sets of 30 s sprints on a cycle-ergometer with 4 min of recovery against the resistance of 0.375 kg/kg of body mass	HIIT
					Control Group	Traditional continuous aerobic exercise	30 min cycling on a cycle-ergometer at 70% of VO_2max_	HIAE
Arikawa 2013 [31]	Sedentary young women	25.3	319/0	16 weeks	Experimental Group	Moderate-to-vigorous aerobic exercise	30 min/per, 5/week, from moderate to 80–85% HRmax	MHAE
					Control Group	Maintaining current active levels	Maintaining current active levels	MCA
Campbell 2020 [32]	Overweight or obese, postmenopausal, sedentary women	60.7	173/0	12 months	Experimental Group	Moderate-intensity aerobic exercise	60–75% HRmax, ≥ 45 min/day, 5 days/week	MHAE
					Control Group	Stretching and relaxation	1/week, 45 min/per, stretching and relaxation; asked to not change exercise habits otherwise	MCA
Duggan 2016 [34]	Overweight or obese, postmenopausal women	57.8	204/0	12 months	Experimental Group	Moderate-to-vigorous aerobic exercise	45 min/day, 5/week, moderate to 70–85% HRmax of aerobic exercise, 2/week of home exercise, 3/week supervised sessions	MHAE
					Control Group	Maintaining current active levels	No change in their diet or exercise habits	MCA
Mallard 2017 [38]	Participants with type 2 diabetes	57.0	14/22	12 weeks	Experimental Group	High-intensity interval training	10 min warm-up at 70% HRmax, 4 × 4 min HIIT at 90–95% HRmax, interspersed with 3 min active recovery at 70% HRmax in RI, ending with 5 min cool-down, 40 min total, 3/week.	HIIT
					Control Group	Moderate-intensity continuous training	210 min/week unsupervised moderate-intensity at 70% HRmax exercise	MIAE
Small 2017 [39]	Patients with stages 3–4 of chronic kidney disease	61.9	59/78	12 months	Experimental Group	Standard nephrology care	A review by a nephrologist recommended lifestyle modification but no specific information or education	MCA
					Control Group	Exercise training and lifestyle intervention	Gym session (20–30 min aerobic training, 20 min resistance training), home-based whole-body resistance training exercises, 2–3/week.	MIAE + RT
Allgayer 2008 [30]	Patients with colorectal cancer	58.6	17/44	2 weeks	Experimental Group	Moderate-intensity exercise program	0.3–0.4 maximal individual aerobic exercise performance, 30–40 min/per	MIAE
					Control Group	High-intensity exercise program	0.5–0.6 maximal individual aerobic exercise performance, 30–40 min/per	HIAE
Wadley 2014 [41]	Rheumatoid arthritis patients	56.0	13/6	12 weeks	Experimental Group	High-intensity aerobic exercise	3/week, 70% VO_2max_ aerobic exercise, 3 sets, 3–4 intervals, 30–40 min total	HIAE
					Control Group	Maintaining current activity	Be advised on the benefits of exercise throughout the same period	MCA

MHAE: moderate-to-high-intensity (or vigorous) aerobic exercise; MIAE: moderate-intensity aerobic exercise; HIAE: high-intensity aerobic exercise; HIIT: high-intensity interval training; RT: resistance training; ST: strength training; MCA: maintaining current lifestyle or maintaining current physical activity and being allowed to add some gentle activities.

**Table 2:** Results of individual studies.

Study	Duration	Reporting Time	Indicator	Unit	Main Results		
					Experimental Group	Control Group	Between Groups
Friedenreich 2016 [35]	12 months	Pre-program 6 months 12 months	Plasma 8-OHDG	ng/L	Almost unchanged in all recording times	Increased insignificantly in all recording times	Without significant difference in all recording times
Vezzoli 2019 [40]	12 weeks	Pre-program Post-program	Plasma PCs *	nmol/mg· protein	Decreased significantly	Almost unchanged	With significant difference
			Urine 8-OHDG *	ng/mg· creatinine	Decreased significantly	Increased insignificantly	With significant difference
Correa 2021 [33]	6 months	Pre-program Post-program	Plasma F2-isoprostanes *	mg/mL	Decreased significantly	Increased insignificantly	With significant difference
Hudson 2008 [36]	1 time	Pre-exercise Post-exercise 60 min post-exercise	Plasma PCs *	umol/mg protein	Increased significantly only 60 min post-exercise	Increased significantly both post-exercise and 60 min post-exercise	With significant difference only post-exercise
Jamurtas 2018 [37]	1 time	Pre-exercise Post-exercise 24 h post-exercise 48 h post-exercise 72 h post-exercise	Plasma PCs *	nmol/mg· protein	Increased significantly only post-exercise	Almost unchanged in all recording times	With significant difference only post-exercise
Arikawa 2013 [31]	4 months	Pre-program Post-program	Plasma F2-isoprostanes	pg/mL	Decreased significantly	Decreased significantly	Without significant difference
Campbell 2020 [32]	12 months	Pre-program Post-program	Urinary F2-isoprostane	mg/mg creatinine	Increased insignificantly	Decreased insignificantly	Without significant difference
Duggan 2016 [34]	12 months	Pre-program Post-program	Plasma F2-isoprostanes	mg/mL	Decreased significantly	Decreased insignificantly	With significant difference
Mallard 2017 [38]	12 weeks	Pre-program Post-program 12 months post-program	Plasma PCs	U/mg protein	Decreased insignificantly in all recording times	Increased insignificantly in all recording times	Without significant difference in all recording times
			Plasma F2-isoprostanes	pg/mL	Increased insignificantly in all recording times	Decreased insignificantly in all recording times	Without significant difference in all recording times
Small 2017 [39]	12 months	Pre-program Post-program	Plasma F2-isoprostanes	pg/mL	Increased insignificantly	Increased insignificantly	Without significant difference
Allgayer 2008 [30]	2 weeks	Pre-program Post-program	Urinary 8-OHDG	ng/mg	Decreased significantly	Increased insignificantly	With significant difference
Wadley 2014 [41]	12 weeks	Pre-program Post-program	Plasma PCs	mmol/mg protein	Decreased insignificantly	Increased insignificantly	Without significant difference

*: lack of original data of outcome measures.

**Table 3:** The probability rank of each intervention for unhealthy participants.

Intervention	Rank 1	Rank 2	Rank 3	Rank 4	Rank 5
HIAE	0.01	0.04	0.41	0.23	0.31
HIAE + RT	0.07	0.05	0.23	0.26	0.39
HIIT	0.59	0.28	0.05	0.04	0.04
MCA	0.03	0.05	0.24	0.44	0.24
MIAE	0.31	0.58	0.07	0.04	0.01

MIAE: moderate-intensity aerobic exercise; HIAE: high-intensity aerobic exercise; HIIT: high-intensity interval training; RT: resistance training; MCA: maintaining current lifestyle or maintaining current physical activity and being allowed to add some gentle activities.

**Table 4:** Results of the confidence assessment.

Comparison	Structure	Number of Studies	Within-Study Bias	Reporting Bias	Indirectness	Imprecision	Heterogeneity	Incoherence	Confidence Rating	Reason(s) for Downgrading
**AE + RT:MCA**	**Mixed**	1	SC	SC	NC	MC	NC	MC	Very low	Reporting bias & Imprecision & Incoherence
**HIAE:MCA**		1	SC	SC	NC	MC	NC	MC	Very low	Reporting bias & Imprecision & Incoherence
**HIAE:MIAE**		1	SC	SC	NC	SC	MC	MC	Very low	Reporting bias & Heterogeneity & Incoherence
**HIIT:MIAE**		4	NC	SC	NC	MC	NC	MC	Very low	Reporting bias & Imprecision & Incoherence
**AE + RT:HIAE**	**Indirect**	0	SC	SC	NC	MC	NC	MC	Very low	Reporting bias & Imprecision & Incoherence
**AE + RT:HIIT**		0	SC	SC	NC	SC	MC	MC	Very low	Reporting bias & Heterogeneity & Incoherence
**AE + RT:MIAE**		0	SC	SC	NC	SC	MC	MC	Very low	Reporting bias & Heterogeneity & Incoherence
**HIAE:HIIT**		0	SC	SC	NC	SC	MC	MC	Very low	Reporting bias & Heterogeneity & Incoherence
**HIIT:MCA**		0	SC	SC	NC	SC	MC	MC	Very low	Reporting bias & Heterogeneity & Incoherence
**MCA:MIAE**		0	SC	SC	NC	SC	MC	MC	Very low	Reporting bias & Heterogeneity & Incoherence

MIAE: moderate-intensity aerobic exercise; HIAE: high-intensity aerobic exercise; HIIT: high-intensity interval training; RT: resistance training; MCA: maintaining current lifestyle or maintaining current physical activity and being allowed to add some gentle activities.

## Data Availability

Not applicable.

## Supplementary Materials

The following supporting information can be downloaded at: https://www.mdpi.com/article/10.3390/antiox11091751/s1, Table S1: The original data of each outcome measure.
